# Differentiation of Wharton’s jelly-derived mesenchymal stromal cells into hepatocyte-like cells using a refined method

**DOI:** 10.1186/s12860-025-00534-y

**Published:** 2025-03-03

**Authors:** Afsoon Afshari, Negar Azarpira, Sara Pakbaz

**Affiliations:** 1 Nephro-Urology Research Center, Shiraz University of Medical Sciences, Shiraz, Iran; 2 Transplant Research Center, Shiraz University of Medical Sciences, Shiraz, 7193711351 Iran; 3 Department of Pathology and Laboratory Medicine, University of Toronto, Toronto, Canada

**Keywords:** Wharton jelly, Hepatocyte-like cells, MicroRNAs, Electroporation, Transfection

## Abstract

**Background:**

The production of functional hepatocyte cells in enough quantities is of paramount importance for the replacement of lost hepatocytes. In this investigation, a series of 7-mimic microRNAs was harnessed to induce the differentiation of Wharton’s jelly-derived mesenchymal stromal cells (WJ-MSCs) into hepatocyte-like cells (HLC) through the application of two distinct techniques: transfection agents and electroporation. The results were then compared with those of HLCs differentiated through the consumption of chemical compounds.

**Results:**

Different time points (48 h, 72 h, and 96 h), unlike concentrations of mimic miRNAs (100 pM, and 200 pM), and dissimilar combinations of mimic-miRNAs (4-mimic and 7-mimic miRNAs) were selected to assess the stage of differentiated cells through electroporation and lipofection methods. For chemical differentiation, a two-step chemical hepatic differentiation protocol was used (for 21 days). The expression level of eleven key genes that were selected to estimate the stage of produced HLCs by each method were tested at different time points, concentrations and combination of mimic-miRNA. Results demonstrated that the 7-miR-mimics/72 h culture method by electroporation, then the 7-miR-mimics/72 h culture method by lipofection, and finally the chemical differentiation (72 h culture) showed the best result for differentiation. Furthermore, the period in which HLCs are maintained under culture conditions is important, as prolonged culture (more than 72 h) leads to cell loss.

**Conclusion:**

In conclusion, the results demonstrated that the 7-miR cocktail delivered by electroporation after 72 h effectively promoted the acquisition of hepatocyte-like characteristics which was evidenced by a significant decrease in the Oct4 stemness factor and an increase in the expression of ALB, TAT, AAT, CYP, G6P and HNF4A.

**Supplementary Information:**

The online version contains supplementary material available at 10.1186/s12860-025-00534-y.

## Introduction

Given the extensive range of functions performed by the liver within the body, many malfunctions in this organ can lead to irreversible damage [1]. Consequently, end-stage liver diseases often necessitate liver transplantation [2]. The critical shortage of liver donors and the lengthy waiting lists for transplant recipients have compelled researchers to explore innovative techniques for differentiating mesenchymal stromal cells (MSCs) into cells resembling hepatocytes (HLCs) [3]. The limitations of primary hepatocyte cultures, including their unsuitability for prolonged culture and significant variability between donors, have been well-documented [4, 5]. While human primary hepatocytes are considered an optimal cellular resource for these objectives, their utilization in pharmaceutical and clinical settings is impeded by limited availability, limited proliferative capacity, and rapid functional deterioration during prolonged in vitro culture periods [6, 7].

MSCs have attracted significant attention in this context due to their relatively straightforward culturing process, their high self-renewal capacity, and their ability to differentiate in multiple directions. These cells can be sourced from various sources, such as placenta, umbilical cord blood, Wharton’s jelly (WJ), bone marrow, and adipose tissue, each with slight variations [8].

Wharton jelly-derived mesenchymal stromal cells (WJ-MSCs) are a valuable source of stem cells that are typically discarded as medical waste following delivery, yet they do not raise any clinical concerns. Furthermore, their high potential for expansion, stable karyotype, immunomodulatory capabilities, and lack of tumorigenicity further establish them as a promising source for differentiation protocols [9].

MicroRNAs (miRNAs, miRs) are short noncoding RNAs, typically 18–22 nucleotides long, renowned for their ability to regulate gene expression [10–12]. Research has extensively examined the role of miRNAs in liver development [13]. For example, miR-122 is highly expressed in the normal liver and is associated with the maturation processes of fetal liver cells [14]. Additionally, other miRNAs, such as miR-106a, -574-3p and − 451, have been identified as linked to differentiation into hepatocyte-like cells (HLCs) [15].

One study identified three key miRNAs (hsa-miR-26b-5p, hsa-miR-148a-3p, and hsa-miR-423-3p) that could improve hepatocyte generation and liver regeneration [16]. Has-miR-424-5p functions as a tumor suppressor microRNA in hepatocellular carcinoma (HCC) by inhibiting cell proliferation through targeting specific genes like E2F7. It blocks cell cycle progression, causing cell arrest in the G0/G1 phase, and reduces cell viability while promoting apoptosis in liver cells. These actions collectively help control the growth of cancerous cells in the liver [17, 18].

miR-542-5p plays a pivotal role in hepatocyte differentiation, with its expression levels dynamically increasing during the differentiation of human umbilical cord mesenchymal stromal cells (hUC-MSCs) and liver-derived progenitor cells (LDPCs). Identified as one of seven microRNAs exhibiting over-expression with at least a four-fold change during hepatic differentiation [19], miR-542-5p displays variable expression patterns across different cell types, contrasting with osteogenic differentiation and hepatocyte cell lines [19]. Beyond hepatic differentiation, miR-542-5p is implicated in other cellular processes, such as the proliferation of osteosarcoma cells [17].

Has-miR-1246 and − 1290 are two of six microRNAs identified as critical for hepatic differentiation of hUC-MSCs. Notably, when overexpressed together with six other specific miRNAs, it can stimulate the conversion of mesenchymal stem cells into functionally mature induced hepatocytes (iHep) [20]. It affects genes involved in proliferation, apoptosis, and metastasis [21].

Increased levels of miR-30a-5p have been found during hepatic differentiation, and its ectopic expression, particularly when combined with miR-122, can activate MSCs conversion [22].

Finally, Zhou et al. identified a set of 7 miRNAs that were differentially overexpressed in HLCs. They used these miRNAs as a novel method to differentiate MSCs into HLCs, aiming to generate functional HLCs for the treatment of liver diseases [23]. By considering the importance and role of selected miRNAs (mir-122-5p, -148a-3p, -424-5p, 542-5p, -1246, -1290, and -30a-5p) in differentiation of HLCs two different mix of the mimic-miRNAs was produced for studying the capacity of miRNAs in differentiation. The first mix composed of all 7-mimic miRNAs and the second was the mix lacking miR-30a-3p, -1290 and -1246.

The method for inducing in vitro maturation of stromal cells into HLCs has been largely described as partially successful. Notably, neither pluripotent nor multipotent cells have ever been reported to fully and functionally mature into human hepatocytes. In other words, despite the extensive exploration of various biochemical differentiating protocols, the resulting HLCs remain immature with limited functional properties. Although the impact of adding biochemicals has been thoroughly investigated, the influence of bioelectrical forces such as electric shocks (electroporation method) on hepatic differentiation remains unknown [24, 25]. Consequently, this study aimed to examine the potential of miRNA-induced hepatic differentiation through electroporation or transfection, in comparison to biochemical-induced hepatic differentiation. Furthermore, to evaluate the quality of differentiated cells, and considering the challenge in the mRNA expression levels of critical genes such as albumin (ALB), Alpha Fetoprotein (AFP), cytokeratin 7 (CK7), cytokeratin 18 (CK18), octamer 4 (Oct4), glucose-6-phosphate (G6P), tyrosine aminotransferase (TAT), alpha-1 antitrypsin (AAT), cytochrome P450 (CYP), hepatocyte nuclear factor 1 A (HNF1A), and 4 A (HNF4A) were evaluated.

## Materials and methods

**Fig. 1:**
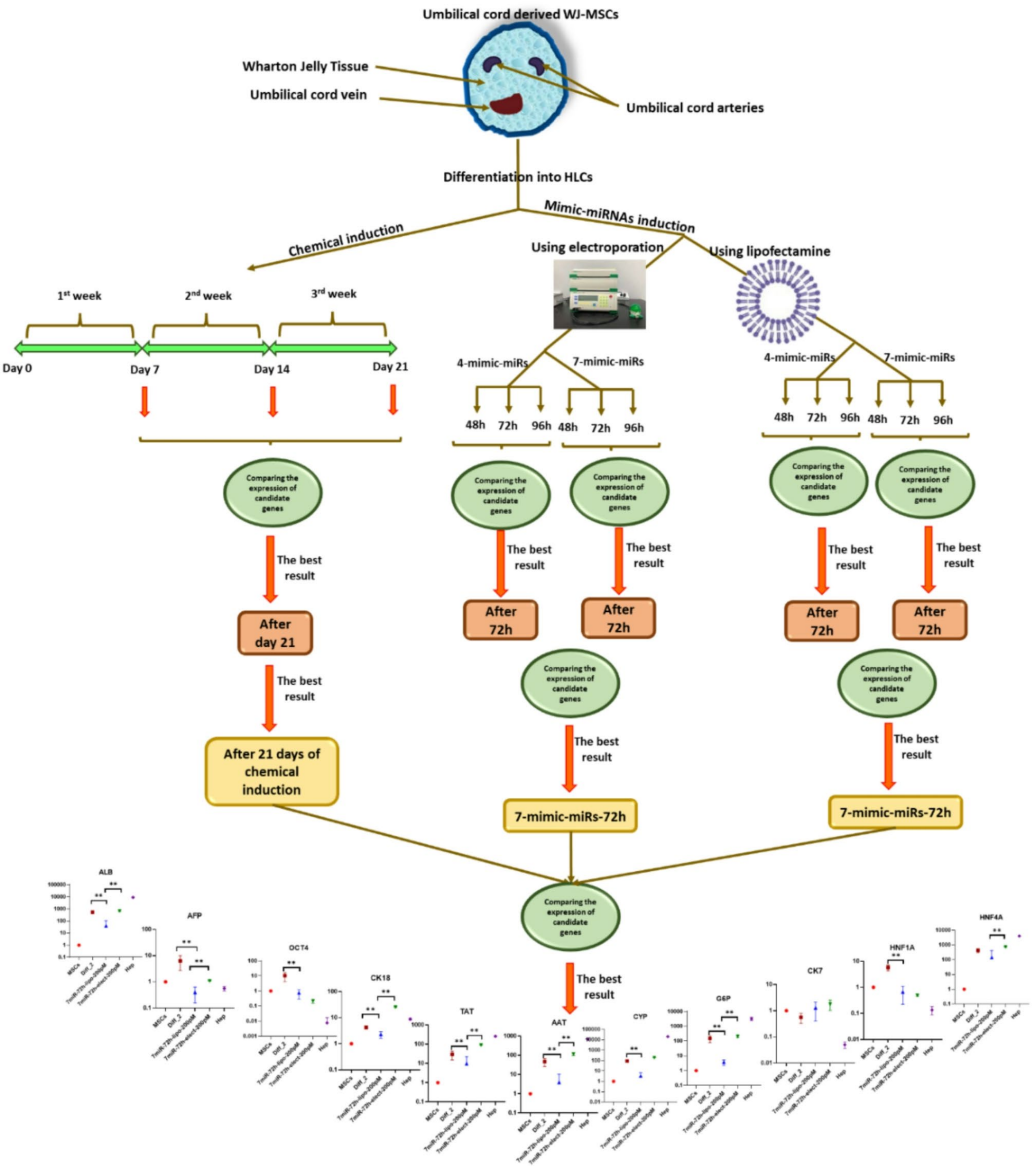
Schematic procedure of the processes. MSCs (mesenchymal stromal cells); Diff_2 (differentiated cells after week 2); Hep (hepatocytes)

All methods were carried out in this research were in accordance with relevant guidelines and regulations and all experimental protocols were approved by Ethics Committee of the Shiraz University of Medical Sciences (Shiraz, Iran). Finally, informed consent was obtained from all subjects and/or their legal guardian(s). Additionally, all the test were done in three replicates in order to obtain more precise results. To enhance the clarity of the procedures utilized in this project, the process is illustrated in Fig. 1.

### Isolation and culture of WJ-MSCs

Nine umbilical cords were obtained from pregnant women at Hafez Hospital (Shiraz, Iran) between 2020 and 2021, after providing informed consent (IR.SUMS.REC.1396.S759). The experimental design of the study followed the guidelines set by the Ethics Committee of the Shiraz University of Medical Sciences (Shiraz, Iran).

Umbilical cords (UCs) were thoroughly washed with phosphate buffered saline (PBS) that contained 5% penicillin/streptomycin to eliminate any residual blood. Subsequently, the UCs were cut into 3–4 cm long pieces, and each segment was longitudinally incised to remove the veins and arteries. The Wharton jelly (WJ) was then cut into 2–3 mm^3^ explant pieces in Petri dishes. The medium, comprising DMEM-F12 (Dulbecco’s Modified Eagle Medium/Nutrient Mixture F-12; GIBCO, USA) supplemented with 10% fetal bovine serum (FBS) (GIBCO, USA) and a 1% solution of penicillin/streptomycin was supplemented to the dishes, which were subsequently incubated at 37 °C in a 5% CO_2_ environment. The medium was changed in a 50%-50% ratio every two days until the expanded cells reached a confluence of 80–90%.

### Proliferative capacity and characterization of isolated WJ-MSCs

Throughout the passages, the WJ-MSCs underwent trypsin (GIBCO, USA) treatment, and after reaching around 80% confluence, they underwent a count using trypan blue. The average population doubling for each passage was ascertained by computing the mean cell counts and applying the formula: PD = (logNt - logN0) / log 2, where Nt denotes the quantity of harvested cells and N0 signifies the initial cell count for each passage [26] (results in S1).

For characterization of isolated WJ-MSCs using flow-cytometry (Becton Dickinson, USA), the cells were collected from culture dishes and washed with phosphate-buffered saline (PBS). Following this, the cells were incubated in a blocking solution comprising 10% goat serum. Subsequently, the cells were exposed to FITC-conjugated anti-CD34 antibodies (a marker for hematopoietic lineage; Biolegend, Cat: 343503), as well as CD90 (Biolegend, Cat: 328108), CD73 (Biolegend, Cat: 344016), and CD105 (Biolegend, Cat: 323204) antibodies as stromal surface markers (all obtained from Abcam, Cambridge, UK) (Figure S1). 4% paraformaldehyde used for fixing cells. In order to mitigate nonspecific staining, isotype control antibodies were appropriately employed (results in S2).

### Potential for adipogenic and osteogenic differentiation of WJ-MSCs

To evaluate the adipogenic differentiation potential of WJ-MSCs, the cells were cultured in DMEM supplemented with human adipogenic stimulatory factors (StemCell Technologies Inc, Canada) for three weeks. Following this incubation period, the cells were stained with oil red to visualize any lipid accumulation, indicative of adipogenic differentiation.

When the cells reached around 80% confluence, the growth medium was substituted with an osteogenic differentiation medium. This medium included DMEM-LG (Invitrogen, Germany) supplemented with 10% FBS, 1% penicillin/streptomycin, 100 nM dexamethasone, 10 nM β-glycerophosphate, 2 nM L-glutamine, and 0.2 mM ascorbate (Sigma, Germany). The cells were then cultured in this osteogenic differentiation medium for 21 days, with the medium changed every 3 days. Subsequently, alizarin red staining was employed to observe calcium deposition in the differentiated cells, indicative of osteogenic differentiation.

### Isolation of adult human primary hepatocytes

Primary adult human hepatocytes utilized in this investigation were obtained from five deceased donors of liver tissue provided by the Hepatocyte Bank of Dr. Hossein Aghdaei (Shiraz University of Medical Sciences). Approval from the Medical Ethics Committee of the Shiraz University of Medical Sciences was obtained prior to the commencement of the study. Informed consent was obtained from the guardians of the donors. The samples were from donors aged between 49 and 67 years (mean = 58.6 years), consisting of 2 women (40%) and 3 men (60%).

The isolation process began with the extraction of hepatocytes using the collagenase perfusion technique. This involved the insertion of cannulas into large hepatic vessels and the flushing of liver tissue with three different perfusion solutions. The cannulated tissue and perfusion tubes were then placed in a sterile organ bag and maintained in a 37 ° C water bath. The first perfusion solution contained calcium and magnesium-free HBSS with ethylene glycol tetra acetic acid (EGTA) and N-acetyl cysteine, while the second solution was HBSS without EGTA. Subsequently, the tissue was digested using collagenase (Sigma, Germany) and DNase (Sigma, Germany). The digested tissue was subjected to mechanical disruption, filtration, and centrifugation. Following washing of the hepatocytes, they were suspended in Williams E medium (GIBCO, Germany) supplemented with various additives. Viability and quantity of hepatocytes were assessed using the trypan blue exclusion test [27]. Most of the cells were alive and attached to the surface of the tissue culture plate. Then, these human primary hepatocytes were used as a positive control in subsequent experiments.

### Chemical hepatic differentiation of WJ-MSCs

In the following, a 2-step protocol was used in order to induce hepatic differentiation. This method is composed of mixing various chemicals and growth factors which here is called chemical hepatic differentiation, briefly. The WJ-MSCs were cultured until they reached 80–90% confluency, after which they were subcultured in a maintenance medium. The medium used in the experiment comprised high glucose DMEM (GIBCO, USA) supplemented with 10% FBS, 1% penicillin/streptomycin, 2 mM L-glutamine (GIBCO, Germany), 10 ng/ml basic fibroblast growth factor (bFGF; Sigma-Aldrich), and 10 ng/ml epidermal growth factor (EGF; Sigma-Aldrich). Cells were seeded at a density of 3 × 10^3^ cells/cm^2^ (approximately 30–40% confluency) in T75 flasks. Once they reached 80–90% confluency, cells were removed from flasks using 0.1% trypsin-EDTA (GIBCO, Germany), centrifuged at 1500 rpm/5 minutes, and washed with PBS. The cells were then cultured at a density of 8 × 10^3^ cells/cm^2^ in preparation for hepatic differentiation. Cells were incubated in an incubator at 37 ° C in a humidified atmosphere containing 5% CO_2_.

Hepatic differentiation was carried out using a 2-step protocol. In Step 1, cells were cultured in a medium comprising DMEM supplemented with 10 ng/ml bFGF, 0.61 g/L nicotinamide (Sigma-Aldrich) and 100 units/ml penicillin, 100 µg/ml streptomycin, 2 mM L-glutamine and final concentration of hepatocyte growth factor (HGF; Sigma-Aldrich) (20 ng/ml in three steps during the week) for 7 days.

In step 2, a maturation medium composed of DMEM supplemented with 20 ng/ml of oncostatin M (Sigma-Aldrich), 1 µmol/L dexamethasone (Sigma-Aldrich) and 50 mg / ml of insulin - transferrin - selenium (6.25 mg/ml insulin, 6.25 mg/ml transferrin, 6.25 ng/ml selenious acid; Sigma-Aldrich) was used for a 2-week induction. The media were changed every 2 days [28].

### MicroRNA-mimic hepatic differentiation using transfection

Initially, Lipofectamine 2000 (Invitrogen, USA) was evaluated for its efficacy using three different experimental setups: (1) HEK293 cells with a GFP-PEG plasmid (4 h and 24 h), (2) WJ-MSCs with a GFP-PEG plasmid (4 h and 24 h), and (3) WJ-MSCs with mimic-miR-FAM (fluorescently labeled with FAM) using fluorescent microscope (CKX53, Olympus, Japan).

Mimics of miR-122, miR-148a, miR-424, miR-542-5p, miR-1246, miR-1290 and miR-30a were synthesized by eurofins Genomics, Germany. Additionally, the same company synthesized a negative control, Mimic-NC. To initiate cell transfection, WJ-MSCs were seeded at a density of 3 × 10^4^ cells/well in 24-well plates. The transfection mixes were prepared by combining mimic miRs at two different concentrations (100 and 200 pmol for each miR) in two distinct combinations: the first mix contained all mimic miRs, while the second mix included all mimic miRs except miR-1246, miR-1290, and miR-30a. Each miRNA combination was diluted in 100µL of OptiMEM (Invitrogen, USA) and thoroughly mixed. Subsequently, 1µL of Lipofectamine 2000 (Invitrogen, USA) was diluted in 100µL of OptiMEM and combined with the miRNA mix. After incubation at room temperature for 15 min, the medium in each well was replaced with the prepared mix for 4 hours. Negative controls (mimic-NC and MSCs without transfection mix) were also included. After 4 hours, the transfection medium was replaced with normal medium (DMEM-F12 supplemented with 10% FBS, 100 units / ml penicillin and 100 µg/mL streptomycin). Samples were collected at 48-, 96-, and 72-hour time points for further analysis, stored in tubes at -70 ° C. The sequences of the mimic miRNAs used are summarized in Table 1.

**Table 1:** The sequence of mimic miRNAs

Mimic-miRs (ID)	Sequence (5’ to 3’)
hsa-miR-122-5p (MIMAT0000421)	UGGAGUGUGACAAUGGUGUUUG
hsa-miR-30a-5p (MIMAT0000087)	UGUAAACAUCCUCGACUGGAAG
hsa-miR-148a-3p (MIMAT0000243)	UCAGUGCACUACAGAACUUUGU
hsa-miR-424-5p (MIMAT0001341)	CAGCAGCAAUUCAUGUUUUGAA
hsa-miR-542-5p (MIMAT0003340)	UCGGGGAUCAUCAUGUCACGAGA
hsa-miR-1246 (MIMAT0005898)	AAUGGAUUUUUGGAGCAGG
hsa-miR-1290 (MIMAT0005880)	UGGAUUUUUGGAUCAGGGA
hsa-miR-NC	NNNNNNNNNNNNNNNNNNNN
Mmu-miR-210-3p-FAM	CUGUGCGUGUGACAGCGGCUG

### Mimic microRNA hepatic differentiation using electroporation

In our study, we compared the transfection potential of WJ-MSCs by modifying various electroporation parameters, including voltage, pulsation mode, and the number of pulses. The most effective transfection was achieved using a 600 V, 0.1 msec, two-pulse square wave setting. Additionally, a Gene Pulser Xcell electroporation instrument (BIO-RAD, USA) was employed for this investigation. Furthermore, all experiments were replicated using different concentrations of the miRNA-mimic complex (100 ng or 200 pmol, comprising 7 or 4 mimic miRs complex) and at varying time intervals (48, 72, and 96 h) in triplicate. As a control, we initially established the experiment using a GFP-PEG plasmid (containing a eukaryotic promoter) and fluorescent-labeled mimic miRNAs in WJ-MSCs.

### RNA extraction, reverse transcription-polymerase chain reaction (RT-PCR) and SYBR green real-time PCR analysis for mRNAs of studied genes

To extract RNA, the samples stored at -70 °C were utilized, and Trizol reagent (Invitrogen; Thermo Fisher Scientific, Inc.) employed for total RNA extraction. To determine RNA quality and quantity, UV spectroscopy used to measure RNA concentration, with an A260 value. Purity is assessed by measuring absorbance at 280 nm for protein contamination, with pure RNA having an A260/A280 ratio of about 2.1. Values between 1.8 and 2.0 were acceptable, and absorbance at 230 nm used to indicate the presence of contaminants like guanidine salts or phenol. Finally, RNA integrity is assessed using 1% gel electrophoresis.

EURx (UK) cDNA synthesis kit and real-time PCR kit were used for cDNA synthesis and assessing the mRNA expression levels of the genes of interest. The specific primer pairs and conditions can be found in Table 2. The thermocycling conditions were as follows: initial denaturation at 95 °C for 30 s, then 40 cycles of denaturation at 95 °C for 15 s, primer annealing at the specified temperature for 20 s, and PCR product elongation at 72 °C for 30 s. The comparative quantification cycle method, 2^−∆∆Ct^, was applied to determine the relative expression levels, and the mRNA expression levels were normalized with GAPDH.

**Table 2:** Sequences of primers used for RT-qPCR

Primer	Sequences (5’-3’)	Fragment length (bp)	Annealing temperature (˚C)
ALB	F-TGAAGGGAAGGCTTCGTCTG R-GGGAAATCTCTGGCTCAGGC	112	60
AFP	F-TGGATTGTCTGCAGGATGGG R-GTTCCAGCGTGGTCAGTTTG	105	60
CK-18	F-GATCATCGAGGACCTGAGGG R-GATCATCGAGGACCTGAGGG	128	60
HNF4α	F-CTTCTTTGACCCAGATGCCAAG R-GAGTCATACTGGCGGTCGTTG	111	60
G6P	F- GGCTGTGCAGCTGAATGTCT R- TGCTGTGGATGTGGCTGAAA	122	60
TAT	F-TCTCTGTTATGGGGCGTTGG R-ACTAACCGCTCCGTGAACTC	138	60
HNF1α	F-AAGACTTCACGCCACCCATC R-GGACTTGACCATCTTCGCCA	128	60
Cyp3A4	F-ACCGTGACCCAAAGTACTGG R-AGCAAACCTCATGCCAATGC	140	60
OCT-4	F- GCAGAAGTGGGTGGAGGAAG R- CACGAGGGTTTCTGCTTTGC	70	60
AAT	F-CTGTCTCCTCAGCTTCAGGC R- CACGAGACAGAAGACGGCAT	71	60
CK-7	F-CATCGAGATCGCCACCTACC R-ATATTCACGGCTCCCACTCC	81	60
GAPDH	F-GGA CTC ATG ACC ACA GTC CA R- CCA GTA GAG GCA GGG ATG AT	119	58

ALB, albumin; AFP, αfetoprotein; CK-18, cytokeratin 18; HNF4α, hepatocyte nuclear factor 4a; G-6P, glucose-6phosphate; TAT, tyrosineaminotransferase; HNF1α, hepatocyte nuclear factor 1a; CYP3A4, cytochrome P450 3A4; OCT-4, POU5F1 POU class 5 homeobox 1; AAT, α1 antitrypsin; CK-7, cholangiocyte marker 7; GAPDH, glucose aldehyde phosphate dehydrogenase; F, forward; R, reverse

### Statistical analysis

The mean ± standard deviation (SD) was utilized to report the findings of a typical experiment were applicable. Group comparisons were carried out using Student’s t-test and one-way analysis of variance, with multiple group comparisons conducted using the student-Newman-Keuls method. Statistical analysis was completed using SPSS 16.0 software (SPSS, Inc., Chicago, IL, USA), with a significance threshold set at *P* < 0.05.

## Results

### Chemical differentiation of WJ-MSCs

**Fig. 2:**
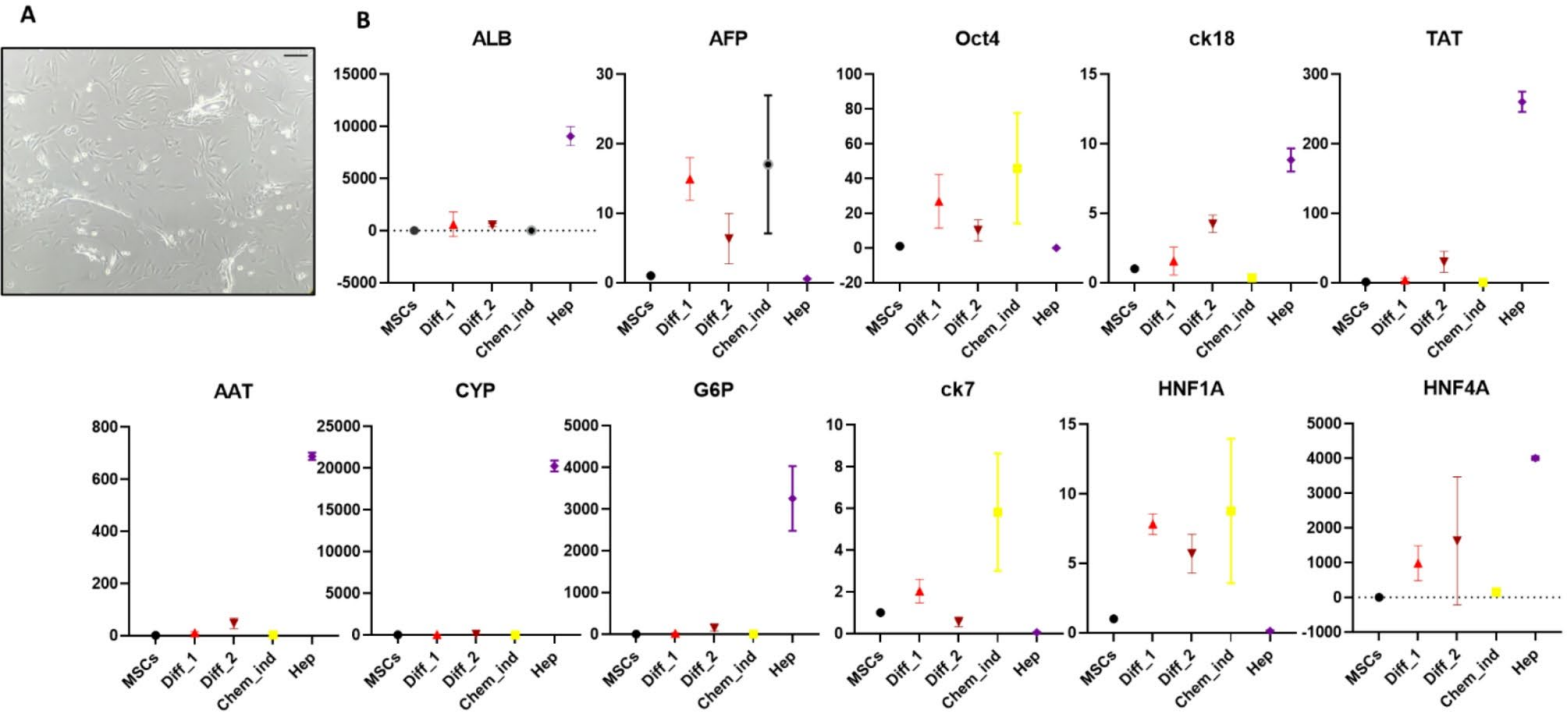
Inducing hepatic differentiation; cells were grown in a medium specifically designed for this purpose, leading to the formation of polygonal hepatocytes and the presence of numerous granules in the cytoplasm; the scale bar is equal to 100 μm (**A**), gene expression differentiated into hepatocytespecific; X-axis represents the different HLCs produced by various methods and The Y-axis is fold change of gene expression (**B**). MSCs (mesenchymal stromal cells); Chem_ind (chemical induction); Diff_1 (differentiated cells after week 1); Diff_2 (differentiated cells after week 2); Hep (hepatocytes)

In order to stimulate the development of liver cells, cells were placed in a specialized medium for hepatic differentiation. Over time, WJ-MSCs began to take on the characteristic polygonal shape of hepatocytes and developed abundant granules within the cytoplasm (see Fig. 2A). The gene expression levels specific to hepatocytes were assessed weekly, revealing a gradual alteration in the expression pattern of these genes in the HLCs following a 3-week exposure to hepatic differentiation medium. Conversely, WJ-MSCs cultured in a standard growth medium were employed as negative controls and exhibited no variations in the designated gene markers (see Fig. 2B).

The results of studying gene expression showed that by moving to the end of the chemical differentiation protocol, it appears that specific hepatocyte genes such as ALB, AFP, TAT, AAT, G6P, HFN1A, ck18, and HNF4A show the pattern which tries to get near the mature human hepatocyte expression pattern. Only CYP3A4, which is related to the late phases of hepatocyte maturation, was not induced even at the end of the chemical induction protocol. Oct4 as a factor that is expressed in stem cells and ck7, which is essential for the development of cholangiocytes, show a gradual decrease as the differentiation protocol proceeds.

### Transfection of WJ-MSCs using microRNA-mimics

**Fig. 3:**
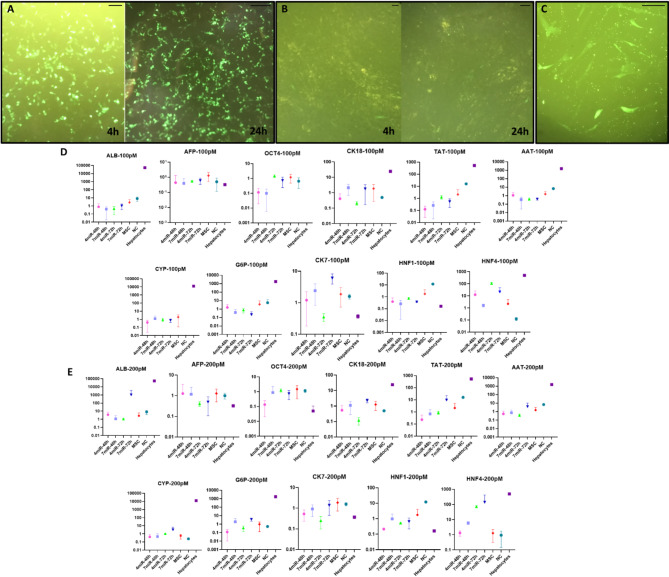
Evaluating the function of Lipofectamine 2000 in different cell types and conditions: HEK293 cells and GFP-PEG plasmid (4 h and 24 h) (**A**), WJ-MSCs and GFP-PEG plasmid (4 h and 24 h) (**B**), and WJ-MSCs and mimic-Mmu-miR-210-3p-FAM (**C**). Evaluating the best conditions of miRNA-mimic complex (7 or 4 mimic-miRs complex) at different concentrations 100 pM (**D**) or 200 pM (**E**) using Lipofectamine 2000 and time points (after 48 h and 72 h). MSCs (mesenchymal stromal cells); Chem_ind (chemical induction); Diff_1 (differentiated cells after week 1); Diff_2 (differentiated cells after week 2); NC (non-coding miRNAs); The asymmetry in the error bars is due to the logarithmic scale used for the Y-axis; X-axis represents the different HLCs produced by various methods and The Y-axis is fold change of gene expression; the scale bars in Fig. 3A-C are equal to 100 μm

To evaluate the function of Lipofectamine 2000 (Invitrogen, USA) tested in different cell types and conditions: HEK293 cells and GFP-PEG plasmid (4 h and 24 h) (Fig 3A), WJ-MSCs and GFP-PEG plasmid (4 h and 24 h) Fig. 3B), and WJ-MSCs plus mimic-Mmu-miR-210-3p-FAM (fluorescently labeled with FAM) (Fig 3C). The results showed the transfecting ability of the agent used.

To find the best concentration of the miRNA-mimic complex (100 pM or 200 pM), the miRNA-mimic complex was tested under different conditions; 7-mimic miRs or 4-mimic miRs complex and at different time points. Better results were shown during the use of a 200 pM concentration of the cocktail of 7-mimic miRNAs. Furthermore, to evaluate the effect of different combinations of mimic miRs (4 miR vs. 7 miR), at different time points (48 and 72 h), the mRNA expression level of all selected genes was evaluated using Real-time PCR (ABI, USA) method (Fig. 3D and E). The result obviously showed that using 200 pM concentrations of the 7-mimic-miRs cocktail after 72 h seems to be a more hepatocyte-like gene expression pattern.

### Transducing WJ-MSCs using MicroRNA mimics via electroporation

For evaluation of the electroporation capacity, the experiment was performed using a GFP-PEG plasmid (contains eukaryotic promoter) and fluorescently labeled mimic miRNA in WJ-MSCs.

**Fig. 4:**
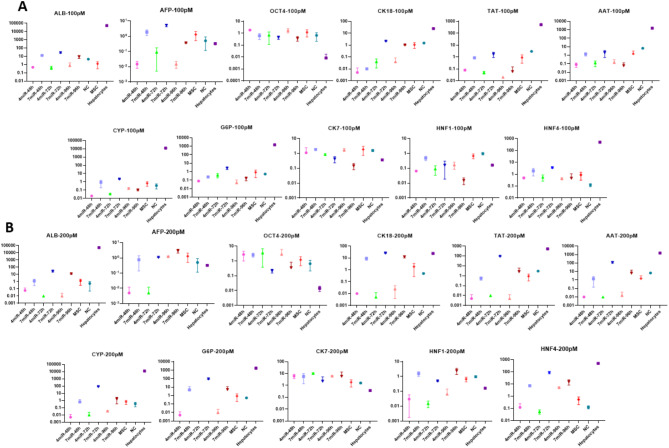
Evaluating of the best conditions of the mimic miRNAs’ complexes (7 or 4 mimic miRs complex) at different concentrations of 100 pM (**A**) or 200 pM (**B**) using electroporation. MSCs (mesenchymal stromal cells); NC (non-coding miRNAs); The asymmetry in the error bars is due to the logarithmic scale used for the Y-axis; X-axis represents the different HLCs produced by various methods and The Y-axis is fold change of gene expression

For finding the best concentration of the miRNA-mimic complex (100 pM or 200 pM, 7 mimic-miRs or 4 mimic-miRs complex), different tests were run each in triplicate. Cells harvested at different time points (48 h, 72 h, 96 h) and the mRNA expression level of all selected genes were evaluated using Real-time PCR method (Fig. 4A and B). Under these conditions, cells exhibited an expression pattern most closely resembling that of the studied genes—specifically AFP, CK18, TAT, AAT, HNF1, and HNF4—similar to hepatocytes used as reference cells, following treatment with a 200 pM concentration of a 7-mimic-miRs cocktail for 72 h (Fig. 4B).

### Comparing the quality of differentiation methods

**Fig. 5:**
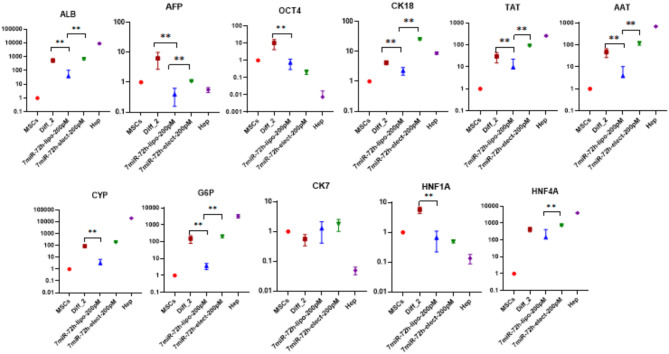
Comparing the quality of differentiation methods for selecting the best method of differentiating WJ-MSCs into HLCs; MSCs (mesenchymal stromal cells); Diff_2 (differentiated cells after week 2); Hep (hepatocytes); The asymmetry in the error bars is due to the logarithmic scale used for the Y-axis

To propose the best method for differentiating WJ-MSCs into HLCs, we compared the results of each method. The best results from each differentiation method were selected for this comparison. This allowed us to evaluate standard chemical differentiation against electroporation and lipofectamine for transducing mimic-miRNAs into cells. The results of this comparison are shown in Fig. 5. The findings indicate that the optimal differentiation condition is achieved using a 7-miR cocktail via electroporation for 72 h. This method appears to produce more mature HLCs due to lower levels of the stemness factor Oct4.

## Discussion

Previous research showed that WJ-MSCs had the ability to express both MSC and ESC markers [29]. These MSCs exhibit the capability to differentiate into all three germ layers [30] and display markers characteristic of endoderm, mesoderm, and ectoderm [31]. Moreover, undifferentiated WJ-MSCs have been observed to express certain hepatocyte markers at a minimal level. Additionally, umbilical cord MSCs have demonstrated the ability to differentiate into hepatocytes with low immunogenicity and functional capacity both in vitro [32, 33] and in vivo [34]. The early expression of liver-specific markers in WJ-MSCs suggests their potential to differentiate into functional hepatocytes, presenting a more viable option compared to stem cells derived from other sources [9]. Given the multitude of diseases that can affect liver function, it is imperative to identify a replacement for these cells. Researchers have proposed that stem cell biology could provide a scalable answer for the treatment of liver disease by providing cells for transplantation and/or cell sources to study liver disorders and identifying toxicity [35, 36]. A rapid and highly effective hepatic differentiation process can be accomplished from human pluripotent stem cells through the utilization of pure small molecule cocktails. This approach offers a cost-efficient framework for studying the molecular underpinnings of human liver diseases in vitro. Stem cells have demonstrated promise for differentiating into HLCs in vivo, with these cells being clinically employed to address liver fibrosis/cirrhosis. Nevertheless, the present stem cell therapy for human liver ailments relies solely on outcomes observed in animal models. The mechanism of action for donor stem/progenitor cells has been predominantly categorized into two main pathways: differentiation into functional cells to substitute damaged cells and secretion of bioactive factors that stimulate the patient’s endogenous tissue-resident progenitor cells [25].

The traditional method for prompting HLC differentiation entails the use of growth factors and entails prolonged induction periods [37]. Nonetheless, this technique is unsuitable for a bio-artificial liver support system (BALSS) that necessitates a rapid generation of a substantial quantity of functional cells. Furthermore, the prohibitive cost of growth factors has restricted the practicality of this approach [38]. Hence, in the present investigation, we aim to introduce a method that closely mimics the molecular expression profile of hepatocytes.

Transduction of miRNAs is crucial for studying their functions, particularly in hepatic maturation. Traditional methods involve the use of synthetic miRNA mimics through transient transfection and viral-mediated gene transduction. In our initial studies, we used Lipofectamine 3000, which demonstrated superior transfection efficiency and potential for enhancing hepatic maturation. However, due to the toxicity of this reagent, the remaining number of cells was insufficient for further analysis (data not shown). Additionally, viral vectors such as lentiviruses offer stable miRNA expression and can infect a wide range of cell types. Nevertheless, potential issues associated with viral vectors led us to exclude them from the current study. There is a risk of viral genes integrating into the host genome when using RNA viruses, which could lead to unwanted genetic modifications and potential safety issues [39]. Additionally, the use of viral vectors requires more complex handling procedures and stricter regulatory compliance, posing challenges for reproducibility and scalability in general research settings [40]. Lastly, we found that conventional transfection methods, such as electroporation or lipid-based transfection, are more straightforward and efficient for the purposes of our study, allowing for better control over transgene expression and reducing the risk of unintended genetic alterations [40]. Although electroporation uses electrical pulses to introduce nucleic acids and may induce cellular stress, it is widely employed due to its simplicity and versatility [41, 42].

An important finding is that not only transcription factors, but also miRNAs have the ability to facilitate cell trans-differentiation [43–45]. In this investigation, we used mimic miRNAs (miR-122-3p, miR-1246, miR-1290, miR-148a, miR-30a, miR-424, and miR-542-5p) previously utilized in the HGF-induced hepatic differentiation model [19, 20].

The proliferative ability of WJ-MSCs, as indicated by cumulative population doublings, is an important factor in their therapeutic potential. The cumulative population doublings calculated in passage 3 provide information on the growth and expansion potential of WJ-MSCs, which is essential for their clinical applications [46].

The findings of this study provide valuable insights into the potential of WJ-MSCs to differentiate into HLCs when cultured in hepatic differentiation medium. The observed morphological changes, such as the formation of polygonal shapes and the presence of granules in the cytoplasm, are indicative of cells taking on the characteristics of hepatocytes.

Furthermore, the study explored the gene expression patterns of specific hepatocyte genes in HLCs, revealing a gradual change in the expression pattern of these genes over the course of the 3-week differentiation period. Up-regulation of genes such as ALB, AFP, TAT, AAT, G6P, HFN1A, ck18, and HNF4A suggests that HLCs were moving toward a more mature hepatocyte expression pattern. However, the low expression of CYP3A4, a gene related to the late phases of hepatocyte maturation, indicates that there may still be some maturation hurdles to overcome [3].

The gradual decrease in the expression of Oct4, a factor expressed in embryonic stem cells, and ck7, essential for cholangiocyte development, further supports the notion that WJ-MSCs were indeed undergoing hepatic differentiation [47].

Investigating the impact of various combinations of mimics (specifically 4miRs versus 7miRs) at different time points (48, 72, and 96 h) clearly demonstrated that the use of a 200 pM concentration of the 7-mimic miRs cocktail after 72 h resulted in a more pronounced hepatocyte-like gene expression pattern. Furthermore, the findings underscore the importance of optimizing the composition and timing of the miR mimic treatments to achieve the desired results in gene expression. This optimization process may involve a careful balance of miR concentrations, combinations, and exposure durations to obtain the most effective and efficient hepatocyte-like gene expression patterns [48].

The comparison of different methods to differentiate WJ-MSCs into HLCs is a critical step in identifying the most effective differentiation strategy [47]. The comparison results, illustrated in Fig. 4, reveal that the optimal differentiation condition is achieved by using the 7-miR cocktail delivered by electroporation, followed by the same miRNA cocktail administered by lipofection after a 72-hour period. This observation suggests that the chemical induction method may confer specific advantages in promoting the differentiation of WJ-MSCs into HLCs, positioning it as a key stage in the acquisition of hepatocyte-like characteristics [49].

Furthermore, the findings of this study validate that traditional culturing techniques can maintain differentiated cell viability for only 72 h, indicating that improved culturing conditions and transitioning from 2D to 3D cultures, such as bioreactors, may be beneficial to maintain differentiated cell viability.

The early genes specific for hepatocytes (AFP, ALB, and HNF4A) are involved in determining cell fate and are regulated by liver-enriched transcription factors. AFP serves as a common indicator of hepatoblasts and fetal hepatocytes, being expressed throughout liver development but suppressed after birth. Additionally, AFP is linked to the liver’s reparative response to diverse injuries, making it a compelling target for investigating differentiation processes [50]. ALB is a typical marker of mature hepatocytes and is involved in the synthesis of plasma proteins [51]. HNF4A is a transcription factor involved in normal liver functions and is required for hepatocyte differentiation and regulation of genes involved in lipid and carbohydrate metabolism [52].

HNF1A has been shown to suppress the innate immune response and inhibit steatohepatitis. The activity of HNF1A in the liver has been linked to the regulation of blood glucose levels and the secretion of atherogenic lipoproteins. HNF1A is a key gene involved in liver development, metabolism, and the pathogenesis of various liver conditions [53].

Induction of TAT and G6P expression is observed during late embryonic liver development, indicating its role in hepatocyte maturation. Additionally, the expression of TAT and G6P is associated with hepatic cell progenitor differentiation and is indicative of the hepatic differentiation process [54]. CYP3A4 is significant in liver development, and is the major adult liver form that plays an important role in late liver development [55].

Oct4, known as POU5F1, functions as a transcription factor that governs the pluripotency and self-renewal of embryonic stem cells (ESCs). Its crucial involvement in liver development and regeneration has been well-established [56]. Research has shown that Oct4 is involved in trans-differentiation of hepatocytes to biliary epithelial cells [57].

In the healthy human liver, hepatocytes commonly exhibit CK18 expression, whereas CK7 and CK18 are predominantly expressed in bile duct cells, with CK7 specifically found in cholangiocytes. CK18 represents the primary intermediate filament protein within the liver [58].

Furthermore, the comparison highlights the potential of the 7-miR cocktail delivered by the electroporation method to produce more mature HLCs, as evidenced by the lower amounts of the stemness factor, Oct4, and the increase of other specific hepatocyte markers such as ALB, AAT, TAT, and HNF4A. This is a crucial aspect of effective differentiation, as the reduction of stemness factors is indicative of a more advanced and specific differentiation process.

Finally, this study suffered from some limitations. Due to the time elapsed since the completion of this project, the quality of our samples has been significantly compromised. The expression levels of most genes and proteins in our differentiated HLCs had diminished over time, making the samples unsuitable for further testing of protein and/or mRNA gene expression of genes involved in distinct liver functions. Consequently, our study was limited to examining the mRNA levels of only 11 genes. While these findings are important, they are insufficient to definitively claim that our differentiation protocol represents an improvement. Future studies should undertake a comprehensive evaluation of gene expression, especially focusing on genes involved in distinct liver functions, to provide a more thorough assessment of the protocol.

## Conclusions

A thorough understanding of the expression patterns of specific markers and the proliferative potential of WJ-MSCs is essential to improve their therapeutic efficacy and clinical significance.

The results of the comparative analysis provide valuable information on the relative effectiveness of distinct differentiation methods for WJ-MSCs. The findings underscore the potential advantages of using the 7-miRs cocktail delivered by electroporation to promote the acquisition of hepatocyte-like characteristics similar to hepatocytes. Furthermore, it is essential to consider the maturity of differentiated cells, as evidenced by the lower amounts of the stemness factor, Oct4, indicating potential advancements in the differentiation process.

Additionally, the duration of maintaining HLCs under culture conditions is a critical consideration, as prolonged culture beyond 72 h can lead to loss of cells. This highlights the necessity of exploring alternative culturing methods following the application of any differentiation approach, in maintaining the viability and functionality of the differentiated cells.

## Electronic supplementary material

Below is the link to the electronic supplementary material.

Supplementary Material 1

## Data Availability

All data generated or analyzed during this study are included in this published article and its supplementary information files.

## References

[1] Takiguchi M. The C/EBP family of transcription factors in the liver and other organs. Int J Exp Pathol. 2002;79:369–91. 10.1046/j.1365-2613.1998.00082.x. 10.1046/j.1365-2613.1998.00082.x PMC3220372 10319019

[2] Dhawan A, Puppi J, Hughes RD, Mitry RR. Human hepatocyte transplantation: current experience and future challenges. Nat Rev Gastroenterol Hepatol. 2010;7:288–98. 10.1038/NRGASTRO.2010.44. 20368738 10.1038/nrgastro.2010.44

[3] Afshari A, Shamdani S, Uzan G, et al. Different approaches for transformation of mesenchymal stem cells into hepatocyte-like cells. Stem Cell Res Ther. 2020;11:1–14. 32033595 10.1186/s13287-020-1555-8 PMC7007672

[4] Rowe C, Goldring CEP, Kitteringham NR, et al. Network analysis of primary hepatocyte dedifferentiation using a shotgun proteomics approach. J Proteome Res. 2010;9:2658–68. 10.1021/PR1001687. 20373825 10.1021/pr1001687

[5] Elaut G, Henkens T, Papeleu P, et al. Molecular mechanisms underlying the dedifferentiation process of isolated hepatocytes and their cultures. Curr Drug Metab. 2006;7:629–60. 10.2174/138920006778017759. 16918317 10.2174/138920006778017759

[6] Hu C, Li L. In vitro culture of isolated primary hepatocytes and stem cell-derived hepatocyte-like cells for liver regeneration. Protein Cell. 2015;6:562–74. 26088193 10.1007/s13238-015-0180-2 PMC4506286

[7] Mitaka T. The current status of primary hepatocyte culture. Int J Exp Pathol. 1998;79:393–409. 10.1046/J.1365-2613.1998.00083.X. 10319020 10.1046/j.1365-2613.1998.00083.x PMC3220367

[8] El-Tantawy WH, Haleem ENA, Al. Therapeutic effects of stem cell on hyperglycemia, hyperlipidemia, and oxidative stress in alloxan-treated rats. Mol Cell Biochem. 2014;391:193–200. 10.1007/S11010-014-2002-X. 24604673 10.1007/s11010-014-2002-x

[9] Borhani-Haghighi M, Talaei-Khozani T, Ayatollahi M, Vojdani Z. Wharton’s Jelly-derived mesenchymal stem cells can differentiate into Hepatocyte-like cells by HepG2 cell line extract. 2015. PMC4359934 25821294

[10] Skrzypczyk A, Kehr S, Krystel I, et al. Noncoding RNA transcripts during differentiation of induced pluripotent stem cells into hepatocytes. Stem Cells Int. 2018;2018. 10.1155/2018/5692840. 10.1155/2018/5692840 PMC6120260 30210551

[11] Sato F, Tsuchiya S, Meltzer SJ, Shimizu K. MicroRNAs and epigenetics. FEBS J. 2011;278:1598–609. 10.1111/J.1742-4658.2011.08089.X. 21395977 10.1111/j.1742-4658.2011.08089.x

[12] Dong H, Lei J, Ding L, et al. MicroRNA: function, detection, and bioanalysis. Chem Rev. 2013;113:6207–33. 10.1021/CR300362F. 23697835 10.1021/cr300362f

[13] Liu D, Fan J, Zeng W, et al. Quantitative analysis of MiRNA expression in several developmental stages of human livers. Hepatol Res. 2010;40:813–22. 10.1111/J.1872-034X.2010.00683.X. 20649821 10.1111/j.1872-034X.2010.00683.x

[14] Doddapaneni R, Chawla YK, Das A, et al. Overexpression of microRNA-122 enhances in vitro hepatic differentiation of fetal liver-derived stem/progenitor cells. J Cell Biochem. 2013;114:1575–83. 10.1002/JCB.24499. 23334867 10.1002/jcb.24499

[15] Khosravi M, Azarpira N, Shamdani S, et al. Differentiation of umbilical cord derived mesenchymal stem cells to hepatocyte cells by transfection of miR-106a, miR-574-3p, and miR-451. Gene. 2018;667:1–9. 10.1016/j.gene.2018.05.028. 29763649 10.1016/j.gene.2018.05.028

[16] Jiang J, Xin J, Ding W, et al. MicroRNA profile of human bone marrow mesenchymal stem cells during hepatic differentiation and therapy. Int J Med Sci. 2022;19:152. 10.7150/IJMS.67639. 34975309 10.7150/ijms.67639 PMC8692113

[17] Zhao Y, Zhu C, Chang Q, et al. MiR-424-5p regulates cell cycle and inhibits proliferation of hepatocellular carcinoma cells by targeting E2F7. PLoS ONE. 2020;15:e0242179. 10.1371/JOURNAL.PONE.0242179. 33201900 10.1371/journal.pone.0242179 PMC7671513

[18] Zhang Y, Li T, Guo P, et al. MiR-424-5p reversed epithelial-mesenchymal transition of anchorage-independent HCC cells by directly targeting ICAT and suppressed HCC progression. Sci Rep 2014. 2014;4(14):1–13. 10.1038/srep06248. 10.1038/srep06248 PMC4150107 25175916

[19] Cui L, Zhou X, Li J, et al. Dynamic MicroRNA profiles of hepatic differentiated human umbilical cord Lining-Derived mesenchymal stem cells. PLoS ONE. 2012;7. 10.1371/journal.pone.0044737. 10.1371/journal.pone.0044737 PMC3440352 22984549

[20] Cui L, Shi Y, Zhou X, et al. A set of MicroRNAs mediate direct conversion of human umbilical cord lining-derived mesenchymal stem cells into hepatocytes. Cell Death Dis. 2013;4. 10.1038/cddis.2013.429. 10.1038/cddis.2013.429 PMC3847311 24232094

[21] Chen S, Fu Z, Wen S, et al. Expression and diagnostic value of miR-497 and miR-1246 in hepatocellular carcinoma. Front Genet. 2021;12:666306. 10.3389/FGENE.2021.666306/BIBTEX. 34163524 10.3389/fgene.2021.666306 PMC8215616

[22] Down-regulation. and clinical role of miR-30a-5p in hepatocellular carcinoma: A study based on public high-throughput datasets| Request PDF. https://www.researchgate.net/publication/316217969_Down-regulation_and_clinical_role_of_miR-30a-5p_in_hepatocellular_carcinoma_A_study_based_on_public_high-throughput_datasets. Accessed 29 Jan 2025.

[23] Zhou X, Cui L, Zhou X, et al. Induction of hepatocyte-like cells from human umbilical cord-derived mesenchymal stem cells by defined MicroRNAs. J Cell Mol Med. 2017;21:881–93. 10.1111/jcmm.13027. 27874233 10.1111/jcmm.13027 PMC5387126

[24] Zhao X, Zhu Y, Laslett AL, Chan HF. (2020) Hepatic differentiation of stem cells in 2D and 3D biomaterial systems. Bioengineering 7. 10.3390/BIOENGINEERING7020047 10.3390/bioengineering7020047 PMC7356247 32466173

[25] Du C, Feng Y, Qiu D, et al. Highly efficient and expedited hepatic differentiation from human pluripotent stem cells by pure small-molecule cocktails. Stem Cell Res Ther. 2018;9:1–15. 10.1186/S13287-018-0794-4/FIGURES/7. 29523187 10.1186/s13287-018-0794-4 PMC5845228

[26] Kern S, Eichler H, Stoeve J, et al. Comparative analysis of mesenchymal stem cells from bone marrow, umbilical cord blood, or adipose tissue. Stem Cells. 2006;24:1294–301. 10.1634/STEMCELLS.2005-0342. 16410387 10.1634/stemcells.2005-0342

[27] Aghdaie MH, Azarpira N, Shamsaeefar A, et al. Effects of different cold preservation solutions. on the Functions of Cultured Isolated Human Hepatocytes; 2020. PMC7724769 33324474

[28] Yen MH, Wu YY, Liu YS, et al. Efficient generation of hepatic cells from mesenchymal stromal cells by an innovative bio-microfluidic cell culture device. Stem Cell Res Ther. 2016;7. 10.1186/S13287-016-0371-7. 10.1186/s13287-016-0371-7 PMC4992324 27542358

[29] Fong CY, Richards M, Manasi N, et al. Comparative growth behaviour and characterization of stem cells from human Wharton’s jelly. Reprod Biomed Online. 2007;15:708–18. 10.1016/S1472-6483(10)60539-1. 18062871 10.1016/s1472-6483(10)60539-1

[30] lo Iacono M, Anzalone R, Corrao S, et al. Perinatal and Wharton’s jelly-derived mesenchymal stem cells in cartilage regenerative medicine and tissue engineering strategies. Open Tissue Eng Regenerative Med J. 2011;4:72–81. 10.2174/1875043501104010072.

[31] Fong CY, Chak LL, Biswas A, et al. Human Wharton’s jelly stem cells have unique transcriptome profiles compared to human embryonic stem cells and other mesenchymal stem cells. Stem Cell Rev Rep. 2011;7:1–16. 10.1007/S12015-010-9166-X. 20602182 10.1007/s12015-010-9166-x

[32] Zhao Q, Ren H, Li X, et al. Differentiation of human umbilical cord mesenchymal stromal cells into low Immunogenic hepatocyte-like cells. Cytotherapy. 2009;11:414–26. 10.1080/14653240902849754. 19513901 10.1080/14653240902849754

[33] Campard D, Lysy PA, Najimi M, Sokal EM. Native umbilical cord matrix stem cells express hepatic markers and differentiate into hepatocyte-like cells. Gastroenterology. 2008;134:833–48. 10.1053/J.GASTRO.2007.12.024. 18243183 10.1053/j.gastro.2007.12.024

[34] Zhang S, Chen L, Liu T, et al. Human umbilical cord matrix stem cells efficiently rescue acute liver failure through paracrine effects rather than hepatic differentiation. Tissue Eng Part A. 2012;18:1352–64. 10.1089/TEN.TEA.2011.0516. 22519429 10.1089/ten.tea.2011.0516 PMC3397120

[35] Zhou W-L, Medine CN, Zhu L, Hay DC. Stem cell differentiation and human liver disease. World J Gastroenterol. 2012;18:2018–25. 10.3748/wjg.v18.i17.2018. 22563188 10.3748/wjg.v18.i17.2018 PMC3342599

[36] Soto-Gutierrez A, Basma H, Navarro-Alvarez N, et al. Differentiating stem cells into liver. Biotechnol Genet Eng Rev. 2008;25:149–64. 10.5661/BGER-25-149. 21412354 10.5661/bger-25-149

[37] Ong SY, Dai H, Leong KW. Inducing hepatic differentiation of human mesenchymal stem cells in pellet culture. Biomaterials. 2006;27:4087–97. 10.1016/J.BIOMATERIALS.2006.03.022. 16616366 10.1016/j.biomaterials.2006.03.022

[38] Mu N, Liu HB, Meng QH, et al. The differentiation of human multipotent adult progenitor cells into hepatocyte-like cells induced by coculture with human hepatocyte line L02. Ann Surg Treat Res. 2015;88:1–7. 10.4174/ASTR.2015.88.1.1. 25553318 10.4174/astr.2015.88.1.1 PMC4279986

[39] Ginn SL, Alexander IE, Edelstein ML, et al. Gene therapy clinical trials worldwide to 2012 - an update. J Gene Med. 2013;15:65–77. 10.1002/JGM.2698. 23355455 10.1002/jgm.2698

[40] Butt MH, Zaman M, Ahmad A, et al. Appraisal for the potential of viral and nonviral vectors in gene therapy: A review. Genes 2022. 2022;13:1370. 10.3390/GENES13081370. 10.3390/genes13081370 PMC9407213 36011281

[41] Jin HY, Gonzalez-Martin A, Miletic AV, et al. Transfection of MicroRNA mimics should be used with caution. Front Genet. 2015;6:154707. 10.3389/FGENE.2015.00340/BIBTEX. 10.3389/fgene.2015.00340 PMC4667072 26697058

[42] Shi B, Xue M, Wang Y, et al. An improved method for increasing the efficiency of gene transfection and transduction. Int J Physiol Pathophysiol Pharmacol. 2018;10:95. 29755642 PMC5943608

[43] Yoo AS, Sun AX, Li L, et al. MicroRNA-mediated conversion of human fibroblasts to neurons. Nature. 2011;476:228–31. 10.1038/NATURE10323. 21753754 10.1038/nature10323 PMC3348862

[44] Jayawardena TM, Egemnazarov B, Finch EA, et al. MicroRNA-mediated in vitro and in vivo direct reprogramming of cardiac fibroblasts to cardiomyocytes. Circ Res. 2012;110:1465–73. 10.1161/CIRCRESAHA.112.269035. 22539765 10.1161/CIRCRESAHA.112.269035 PMC3380624

[45] Shenoy A, Blelloch R. MicroRNA induced transdifferentiation. F1000. Biol Rep. 2012;4:3. 10.3410/B4-3. 10.3410/B4-3 PMC3270586 22312415

[46] Margossian T, Reppel L, Makdissy N, et al. Mesenchymal stem cells derived from Wharton’s jelly: comparative phenotype analysis between tissue and in vitro expansion. Biomed Mater Eng. 2012;22:243–54. 10.3233/BME-2012-0714. 22785368 10.3233/BME-2012-0714

[47] Panta W, Imsoonthornruksa S, Yoisungnern T, et al. Enhanced hepatogenic differentiation of human Wharton’s jelly-derived mesenchymal stem cells by using three-step protocol. Int J Mol Sci. 2019;20. 10.3390/ijms20123016. 10.3390/ijms20123016 PMC6627410 31226809

[48] Raut A, Khanna A. Enhanced expression of hepatocyte-specific MicroRNAs in valproic acid mediated hepatic trans-differentiation of human umbilical cord derived mesenchymal stem cells. Exp Cell Res. 2016;343:237–47. 10.1016/J.YEXCR.2016.03.015. 27001466 10.1016/j.yexcr.2016.03.015

[49] Kim DW, Staples M, Shinozuka K, et al. Wharton’s jelly-derived mesenchymal stem cells: phenotypic characterization and optimizing their therapeutic potential for clinical applications. Int J Mol Sci. 2013;14:11692–712. 23727936 10.3390/ijms140611692 PMC3709752

[50] Xie Z, Zhang H, Tsai W, et al. (2008) Zinc finger protein ZBTB20 is a key repressor of alpha-fetoprotein gene transcription in liver. 10.1073/pnas.0800647105 PMC2504784 18669658

[51] Choi JS, Jeong IS, Park YJ, Kim SW. HGF and IL-10 expressing ALB::GFP reporter cells generated from iPSCs show robust anti-fibrotic property in acute fibrotic liver model. Stem Cell Res Ther. 2020. 10.1186/s13287-020-01745-0. 11:. 32746905 10.1186/s13287-020-01745-0 PMC7398392

[52] Huck I, Matthew Morris E, Thyfault J, Apte U. Hepatocyte-Specific hepatocyte nuclear factor 4 alpha (HNF4α) deletion decreases resting energy expenditure by disrupting lipid and carbohydrate homeostasis. Gene Expr. 2021;20:157. 10.3727/105221621X16153933463538. 33691903 10.3727/105221621X16153933463538 PMC8201658

[53] Tian JM, Schibler U. Tissue-specific expression of the gene encoding hepatocyte nuclear factor 1 May involve hepatocyte nuclear factor 4. Genes Dev. 1991;5:2225–34. 10.1101/GAD.5.12A.2225. 1748280 10.1101/gad.5.12a.2225

[54] Hamazaki T, Iiboshi Y, Oka M, et al. Hepatic maturation in differentiating embryonic stem cells in vitro. FEBS Lett. 2001;497:15–9. 10.1016/S0014-5793(01)02423-1. 11376655 10.1016/s0014-5793(01)02423-1

[55] He H, Nie YL, Li JF, et al. Developmental regulation of CYP3A4 and CYP3A7 in Chinese Han population. Drug Metab Pharmacokinet. 2016;31:433–44. 10.1016/J.DMPK.2016.08.008. 27727071 10.1016/j.dmpk.2016.08.008

[56] Park MR, Wong MS, Araúzo-Bravo MJ, et al. Oct4 and Hnf4α-induced hepatic stem cells ameliorate chronic liver injury in liver fibrosis model. PLoS ONE. 2019;14. 10.1371/journal.pone.0221085. 10.1371/journal.pone.0221085 PMC6690533 31404112

[57] Liu HL, Tang HT, Yang HL, et al. Oct4 regulates the transition of Cancer Stem-Like cells to tumor Endothelial-Like cells in human liver Cancer. Front Cell Dev Biol. 2020. 10.3389/fcell.2020.563316. 8:. 33102474 10.3389/fcell.2020.563316 PMC7554317

[58] Lai YS, Cheng CC, Lee MT, et al. The prognostic value of cytokeratin and sal-like protein 4 expression in hepatocellular carcinoma and intra-hepatic cholangiocarcinoma in Taiwan. Int J Med Sci. 2018;15:1746–56. 10.7150/ijms.28440. 30588199 10.7150/ijms.28440 PMC6299409

