# Supplementary material for: Differentiation of Wharton’s jelly-derived mesenchymal stromal cells into hepatocyte-like cells using a refined method
Source: BMC Mol Cell Biol. 2025 Mar 3;26:9. doi: 10.1186/s12860-025-00534-y (PMC11874389; doi:10.1186/s12860-025-00534-y)
Supplement: Supplementary file 1 — Supplementary Material 1 [file 12860_2025_534_MOESM1_ESM.docx]

**S1- Proliferative ability of WJ-MSCs:** The WJ-MSCs showed adherence and demonstrated elongated spindle-shaped morphology. The cumulative population doublings of WJ-MSCs were calculated by tracking the total number of cell divisions from the initial passage to the third passage. The population doubling time indicates the duration required for the population to double in size. At passage 3, cumulative population doublings of WJ-MSCs were measured at 1.65±0.05.

**S2- WJ-derived MSCs phenotype analysis:** The WJ-MSCs could express CD90, CD105, and CD73; nevertheless, the proportions of cells expressing CD34 were minimal (Figure **S1A-D**).

Histochemical staining further demonstrated the multipotent nature of the MSCs. In the presence of osteogenic and adipogenic media, they exhibited the ability to differentiate into osteoblasts and adipocytes, as depicted in Figures **E** and **F**, respectively.


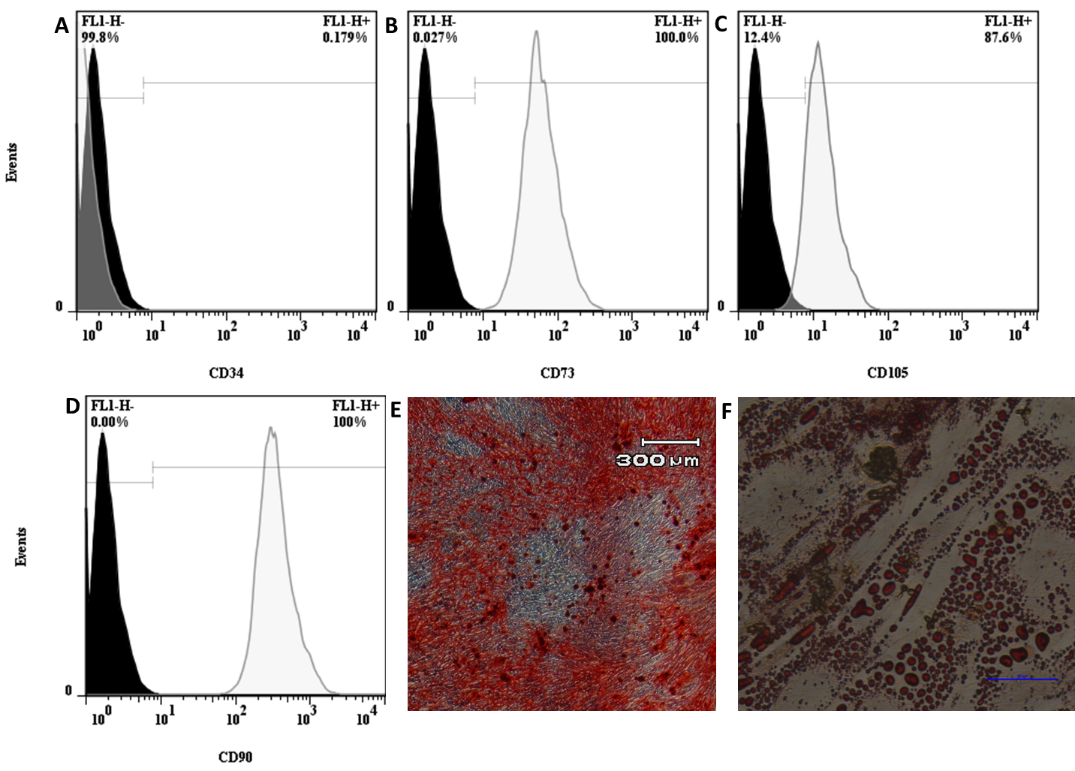


**Figure S1-** WJ-MSCs express a low level of CD34 (A) and a high level of CD73 (B) and CD105 (C) and CD90 (D) and can be differentiated into osteoblasts (E) and adipocytes cells (F).
